# Feasibility, acceptability and efficacy of non-invasive application of chlorhexidine dental coating for seniors in the community: a randomized controlled study

**DOI:** 10.2340/aos.v84.44126

**Published:** 2025-08-07

**Authors:** Heidi Sveistrup, Irene Elizabeth DeHaan, Andrea Pepe, Howard C. Tenenbaum, Anton Svendrovski, Brenda Turgeon, Ross Perry

**Affiliations:** a Faculty of Health Sciences, University of Ottawa, Ottawa, ON, Canada; b Bruyère Health Research Institute, Ottawa, ON, Canada; c iSmile Mobile Dental, Ottawa, ON, Canada; d University of Toronto, Faculty of Dentistry and Faculty of Medicine, Toronto, ON, Canada; e UZIK Consulting Inc, Toronto, ON, Canada; f CHX Technologies, Toronto, ON, Canada

**Keywords:** Chlorhexidine coating 10%, prevention, oral health, barriers to care, oral inflammation, bleeding on probing

## Abstract

**Background:**

Poor oral health (caries and periodontal disease) in seniors is a health risk with minimal preventive care approaches evaluated. The objectives of this study were to: (1) document the oral health status of a sample of Canadian seniors; and then (2) evaluate feasibility, acceptability and efficacy of non-invasive application of chlorhexidine for preventive oral health in the community.

**Methods:**

Dental examinations of 105 Ottawa seniors were followed by a randomized controlled study of 55 seniors with ≥ 16 natural teeth and ≥ 12 sites of bleeding on probing (BOP). Four treatments of either a chlorhexidine 10% w/v (active) or purified water (placebo) were applied over a 9-week period. Study sites included a hospital, seniors’ community centre, medical practice and long-term care facility. Treatments were applied by a hygienist or a nurse in 10–15 minutes, without dental cleaning or scaling. The primary endpoint was change in oral inflammation (BOP).

**Results:**

The sample had more decayed, missing or filled teeth (DMFT) and more periodontal disease than reported in the 2007–2009 Canada Health Measures Survey. BOP was reduced almost 2-fold more in the active group compared to placebo although significant in both groups (active: 7.91%, *p* < 0.001; placebo: 4.1%, *p* = 0.018). The difference between groups in reducton of BOP failed to reach significance (*p* = 0.06) likely due to early stoppage of recruitment.

**Conclusion:**

A four-dose application of chlorhexidine significantly decreased oral inflammation as measured by BOP. The program of non-invasive preventive care in community settings is feasible, clinically impactful, and well received.

## Introduction

The Ottawa Seniors Oral Health Study is the first survey by a dental examiner of seniors’ oral health in 15 years [1] and is the first-ever controlled study of non-invasive preventive dental care delivered where Canadian seniors live or spend their time. The study commenced in late 2022 and concluded in mid 2023. It was initiated for two reasons: (1) to develop and test a new, suitable, and barrier-free model of community-based preventive dental care using chlorhexidine with seniors and (2) to inform the Government of Canada of the oral health status of a sample of seniors, as the Government developed its Canada Dental Care Plan for Seniors [2].

### Oral health needs among Canadian seniors

There is a very large and growing unmet need to improve access to dental services in Canada with an estimated 9 million seniors and adults with disabilities, who have limited ability to receive dental services [3]. A closer analysis of the Canadian population suggests that there may be many more people who face barriers in accessing appropriate dental care services. There are 4 million frail and pre-frail seniors who have difficulty getting to and from the dentist [4, 5]. There are 1.3 million adults receiving homecare services who cannot readily get dental care [6], 1.8 million with severe/very severe disabilities [5, 7], and an estimated 3.6 million Canadian adults with dental fear and anxiety [8].

### Challenges with existing dental care models

Likewise, dental professionals face difficulties delivering care outside of a dental practice because equipment is bulky to transport, and invasive procedures generate aerosols. Existing dental models limit the numbers of patients who can be treated in the community. Minimally invasive approaches such as atraumatic restorative treatment (ART) have been shown to be useful for treating tooth decay in children [9, 10] as well as home-bound elders and people living in residential and nursing homes [11, 12]. ART requires a skilled dentist and is often difficult to implement due to the labour required and low remuneration available. As a minimally invasive dentistry approach, ART has been used to treat tooth decay including root caries. However, there does not appear to be evidence that the approach is effective in the treatment of dysbiosis or periodontal disease. In addition, the use of ART is limited to a dental practice and cannot be implemented by a dental hygienist or nurse.

### Rationale for a new community-based preventive approach

These two realities suggest that a new model of preventive dental care is needed to deliver oral healthcare to Canadian adults in non-dental settings. This model must reduce chair time, require minimal if any dental equipment, be non-invasive, and be able to serve many patients in a day. Moreover, given the huge scale of the access problem, new models of dental care will benefit by including other health-care professionals.

Health Canada approval for a high-strength, sustained-release 10% chlorhexidine coating for the reduction of root caries in high-risk adults was issued in 2004 and the first commercial sale of this preventive coating to Canadian dental professionals occurred in 2007. Since its approval (DIN02046245), this coating has been used by Canadian dental professionals to prevent root caries which is common in older adults and which co-exists with periodontal disease [13]. More recently, dentists and hygienists have used this product off-label for the prevention of periodontal disease [14]. Periodontal disease and caries result primarily from oral dysbiosis which happens both before and after dental treatment [15]. Oral dysbiosis is an over-population of pathogenic bacteria in the dental plaque which emerges as a result of aging, limited oral hygiene, type 2 diabetes, multiple co-morbidities, polypharmacy, frailty and muscular or cognitive disorders.

Some Canadian dental hygienists, dentists, doctors and nurses have also been innovating with the delivery of this coating to treat patients with type 2 diabetes, dementia, special needs, receiving homecare services and with muscular disorders which limit oral hygiene [16]. For example, just prior to the pandemic, a pilot study of this product was successfully conducted by a dental hygienist in a family medical practice to treat chronic oral inflammation in type 2 diabetics during their quarterly medical consultation [17]. This experimental study led to the development of rapid application methods that minimized the time and equipment needed and expanded the venues in which it could be delivered. The rapid procedure involves the direct application of the coating to the full dentition up to and including the gum line in about 10 minutes without a dental prophylaxis or periodontal scaling. In a prospective, pilot observational study of 10 consenting older adults (mean age 72 years) conducted in a Canadian hygiene practice, significant reductions in all stages of periodontal disease were reported with 2 coating applications over 2 weeks. [In an email from Julie DiNardo, RDH (julie@gleamsmile.ca) in May 2022]

In spring 2022, when the Government of Canada announced its plan for a new national dental plan for seniors and people with disabilities, there was little information on the current oral health status of seniors using standard indicators such as decayed, missing and filled teeth (DMFT), periodontal prevalence and functional dentition. Accordingly, the Canadian Dental Hygienists Association asked the product manufacturer of the coating if it could provide data from a dental examination of seniors’ mouths. Similarly, in early 2022, there was no published model for the delivery of an affordable, effective, and appropriately portable and non-invasive preventive dental care service suitable for millions of Canadians with limited access to dental services.

This study responded to the evidence gap by conducting a survey of the oral health of local seniors living in the community, in independent living residences and in longterm care homes, and implemented a protocol for a prospective randomized controlled clinical trial of the rapid application technique involving 10% chlorhexidine coating in consenting seniors living in the community. The aim of this study was twofold: (1) to document oral health in a group of seniors; and (2) to provide preliminary evidence of the feasibility and effectiveness of chlorhexidine dental coating applied non-invasively in community settings.

## Methods

### Study design and population

This was a randomized, double-blind, placebo-controlled trial approved by a hospital Research Ethics Board (#M16-22-052; Bruyère Health, Ottawa Canada), authorized by Health Canada (Clinical Trial Authorization #270250), and registered in the ISRCTM database (ISRCTN14373079). The study was conducted for a period of 10 months (November 2022–August 2023) in three locations in Ottawa: a community seniors’ centre, a long-term care home and a hospital clinical trials assessment site. Participants were recruited by a research staff member from: a roster of community-dwelling seniors interested in clinical research, seniors attending programming in a seniors’ center, seniors living in an independent-living village, and seniors living in a long-term care home. All participants or their substitute decision maker provided written, informed consent. Recruitment ended in June 2023 in order to meet the sponsor’s deadline (September 2023) to submit results to the Government of Canada.

The target population consisted of individuals over 65 years of age with 16 or more natural teeth and 12 or more bleeding on probing (BOP) sites. Individuals (1) with active caries requiring restoration, (2) with severe periodontal disease requiring significant intervention, (3) undergoing periodontal care, (4) with known allergies to the ingredients in the study medication, (5) taking anti-inflammatory medications or other medications for periodontal disease, (6) taking antibiotics for oral abscesses, oral pain, or taking antibiotics for more than 14 days, (7) with uncontrolled epilepsy, (8) with a gag reflex, (9) having behavioral disorders impeding study participation, (10) enrolled in another drug trial, (11) unwilling or unable to personally complete informed consent or provide consent through a substitute decision maker were excluded.

One hundred and five older adults consented and were screened for oral hygiene habits, medical history and three measures of frailty. A comprehensive oral examination documented standard periodontal measures: BOP for early-stage periodontal disease, number of natural teeth, history of dental decay (DMFT), and number of caries lesions. The oral examination of both hard and soft tissues was conducted by an independent hygienist with over 20 years of experience working with seniors in the community using standard methods for measuring decayed teeth, missing teeth and filled teeth. DMFT data were collected both at the start and end of the study to compare caries prevalence in the study versus a national survey conducted in 2007(1) to evaluate if seniors’ oral health had improved, stabilized or deteriorated over time. DMFT was not a study endpoint.

Fifty-five screened and eligible participants consented to the randomized control trial and were randomly assigned to a placebo or active group. Randomization was conducted in blocks of eight study participants. Random numbers were generated for each block of eight with the highest four numbers per block assigned to placebo and the lowest four numbers per block assigned to active. The study participants, assessing hygienist and treating hygienist and nurse were blinded to the intervention assignment.

### Investigational product(s) and instructions

**Figure 1:**
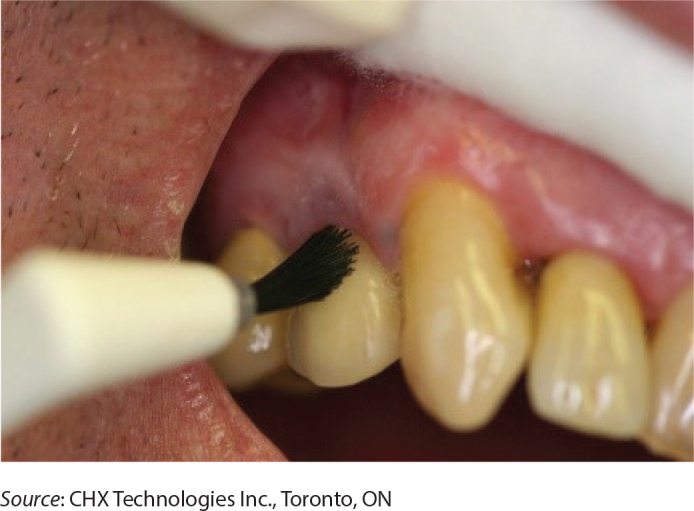
Application of a chlorhexidine coating to the full dentition up to and including the gum line. Identical application procedures were used for the application of the active and placebo products.

Both groups received treatments in the same venues as screening. A blinded hygienist or a nurse applied the study product (placebo or active) using a rapid procedure without dental prophylaxis or periodontal scaling. This procedure was developed and provided to study staff by Julie DiNardo [email from Julie DiNardo, RDH (julie@gleamsmile.ca) May 2022] (Figure 1). Clinical application of the active product involves two stages. An initial coating of the active product is applied to the full dentition up to and including the gum line using a hygienist’s mini brush. After 15 s, a second coating of a binding product is applied over the active product and allowed to air-dry. Salivary flow over the teeth receiving the coating was temporarily blocked by a 2 × 2 gauze held by the provider while applying the active and binding products. The application of the placebo product was identical to the application of the active product and binding product. Participants in both groups were instructed not to brush the teeth for one day and not to floss the teeth for 3 days following application.

The study medication was labelled so neither the hygienist nor the participant could tell whether it was active or placebo. Because the participants had never received the CHX coating before, they had no way of knowing if they received the active or placebo product. Indeed, the participant surveys at the end of the study show that the tolerability of both active and placebo products were were very similar. In terms of blinding the examiner, it is noted that the examiner did not apply the study medication and had no contact with the hygienist or nurse applying the study medications. The examiner also had no knowledge of any participant reactions to the study medication (e.g. bitter taste) as the study coordinator kept this information from the examiner.

The active study medication consisted of 10% chlorhexidine, 20% Sumatra benzoin and absolute ethanol qt (CHX Technologies). Approximately 300 to 500 mL was applied per treatment. A coating of methacrylate was applied in the same manner immediately after the application of the chlorhexidine coating. For the placebo group, purified water was aliquoted into vials identical (size, labeling, color) to those holding the active and binding products. The only identifier on active and placebo product vials was the randomization number. In between treatments, product vials were stored in a temperature-controlled, locked study refrigerator.

### Study visits

At baseline, a research staff member documented demographic data including inclusion and exclusion criteria. Comprehensive oral exams were performed by a single blinded licensed assessing hygienist with over 20 years of experience in providing periodontal care, including 4 years of independent practice with older adults. In some studies of periodontal disease, calibration of examiners might be done. However, in this particular case, we had a single highly experienced examiner who was blinded to treatment. Hence, systematic errors were distributed equally in both groups. Moreover, calibration presumes that there is ‘gold standard’ for probing, but this is not the case [18]. Even in dental undergraduate educational programs, the accuracy of periodontal probing improves to a degree that is acceptable [19]. Finally, DMFT was not an end point variable and is included solely as a comparator to the 2007 Canadian Health Measures Survey [1].

Eligible individuals were immediately recruited to the trial and met with either the treating hygienist or nurse in a separate room. Consented and enrolled participants were randomized by the research staff member to either the active or placebo group, and an initial product application was completed. Following the baseline application, participants were scheduled for repeat applications at weeks 2, 4 and 6 as per the Health Canada-approved treatment protocol. An end-of-study oral exam was scheduled with the assessing hygienist between 4 and 6 weeks following the final application. At the end-of-study visit, in addition to repeating a comprehensive oral exam, participants were asked to respond to a questionnaire documenting the following information: acceptability of the procedure, the importance of the procedure to the patient, willingness to continue with this treatment plan, and the participant’s perception of the impact of the procedure on the health of the gums and general health.

The study team documented factors related to the feasibility of delivering preventive dental care in the community including: safety, the use of a nurse for product application, the time required to deliver care, and ease of delivery in a seniors’ community center, a medical practice, a hospital and in long-term care. All self-reported and whole-body adverse events were recorded.

### Analytical plan

Since there are no studies of individuals with similar characteristics, we used information from a clinical partner [in regular correspondence with Julie DiNardo, RDH; January 2013 to May 2024] to obtain effect size and the G*Power tool to determine sample size. DiNardo reports that 60% of similar patients in her clinical practice showed improvements in BOP post-treatment with the study product. We made a further assumption that in a control group, there would be half of this effect size (30% of patients with improvement). We set statistical power to 80%, level of significance at 0.05, resulting in a sample size estimation of 66 patients in total (33 per group).

Descriptive statistics (e.g. means, frequencies) of the demographic characteristics were summarized for each study group. The primary outcome variable was the number of BOP sites with efficacy determined as the change from the baseline to the end-of-study oral exam within as well as between groups. Data were assessed using the paired sample and independent sample t-test (one-tailed, 5% significance level) on the intent-to-treat study population using last-observation-carried forward to impute any missing data. Baseline comparison between groups was performed using independent samples t-test (or Mann-Whitney test for non-parametric data), and Chi-squared test (or Fisher’s exact test for small counts).

## Results

**Figure 2:**
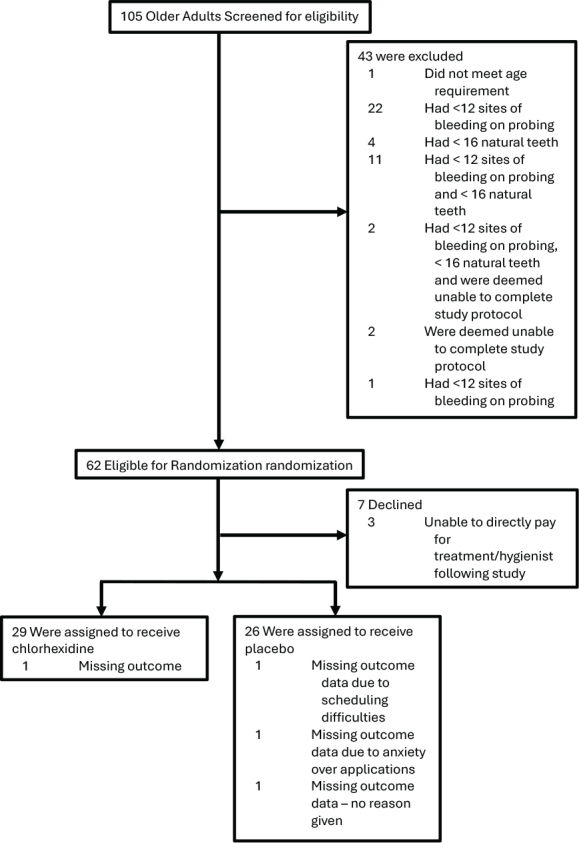
Study flow diagram.

One-hundred and five seniors with a mean age of 78 years were screened (Figure 2). Most were female (56%) and 71% lived independently in the community with 13% in assisted living and 16% in long-term care. A mean of 5 prescription drugs were being taken daily. One in three participants said they felt exhausted in the past week which points to a substantial degree of frailty in this population.

Most seniors reported good daily oral hygiene habits and claimed visiting the dentist at least once a year. A majority showed good knowledge of the importance of having good oral health for maintenance of overall health. Baseline demographics, however, documented significant levels of both dental decay and periodontal disease (Table 1). About 75% of all teeth were decayed, missing or filled, and more than half of participants had periodontal disease. One in five had less than a functional dentition (20 teeth) which is required to chew food and maintain nutrition.

**Table 1:** Key indicators of oral health at baseline with comparison data from the 2007–2009 Canada Health Measures Survey.

Indicator	Ottawa 2022–2023 *N* = 101	Canada Health Measures Survey 2007–2009(1)
Decayed, Missing and Filled Teeth (DMFT) (Mean ± SD)	24 ± 6.5 (Min-Max = 8–48)	16
Prevalence of periodontal disease	*56%	33%
Number of natural teeth (Mean ± SD)	22.96 ± 6.32 (Min-Max = 0–32)	21

* Prevalence of periodontal disease defined as the % of sample with BOP ≥ 12 and 10 PPD ≥ 4 to 5 mm (includes early and moderate stage perio).

### Randomized controlled trial

Forty-three individuals screened at baseline did not meet the inclusion criteria for the RCT. An additional seven declined participation. Fifty-five screened individuals met the inclusion criteria and consented to the randomized controlled trial (Table 2). There were no differences at baseline in the demographic, behavioral and medical risk factors for poor oral health, and for BOP between the active and placebo groups.

**Table 2:** Baseline demographics and clinical characteristics.

Characteristic	Total sample (*n* = 55)	Placebo (*n* = 26)	Active (*n* = 29)	*p*
Age, years				0.349^t^
Mean (SD)	77.58 ± 7.46	76.58 ± 6.42	78.48 ± 8.29	
Min-Max	65 – 96	65 – 90	66 – 96	
Sex				0.721^c^
Female	31 (56%)	14 (54%)	17 (59%)	
Male	24 (44%)	12 (46%)	12 (41%)	
Residing location				0.943^c^
Independent living	39 (71%)	19 (73%)	20 (69%)	
Assisted living	7 (13%)	3 (12%)	4 (14%)	
Long-term care	9 (16%)	4 (15%)	5 (17%)	
Wheelchair	10 (18%)	4 (15%)	6 (21%)	0.733^f^
Dresses self	45 (82%)	21 (81%)	24 (83%)	1.00^f^
Exhausted	19 (35%)	8 (31%)	11 (38%)	0.577^c^
Smoking				0.172^f^
Current smoker	1 (2%)	0	1 (4%)	
Former smoker	22 (40%)	8 (31%)	14 (48%)	
Non-smoker	32 (58%)	18 (69%)	14 (48%)	
Drinking				0.580^c^
Regular	16 (29%)	9 (35%)	7 (24%)	
Occasional	14 (26%)	7 (27%)	7 (24%)	
Never	25 (45%)	10 (38%)	15 (52%)	
# of teeth				0.127^t^
Mean (SD)	24.98 ± 3.44	25.73 ± 3.13	24.31 ± 3.61	
Min-Max	17 – 32	18 – 32	17 – 32	
Median	25	26	25	
BOP sites (#)				0.926^m^
Mean (SD)	36.42 ± 24.96	37.12 ± 28.06	35.79 ± 22.32	
Min-Max	14 – 125	14 – 125	15 – 94	
Median	26	26	27	
BOP sites (%)				0.768^m^
Mean (SD)	24.41 ± 16.65	23.97 ± 17.45	24.80 ± 16.20	
Min-Max	9 – 83	9 – 83	9 – 78	
Median	19	19	19	

BOP: bleeding on probing.

Note: Baseline descriptive values reported as Mean ± SD [range] or frequency (%).

*p* value for the between group comparison:

t Independent samples t test;

c Chi-squared test;

f Fisher’s exact test;

m Mann-Whitney test

Four participants did not complete the study. An additional four participants in long-term care (4/9) had extreme oral inflammation which made assessment of BOP unreliable since bleeding after probing was so excessive that it prevented assessment of adjacent test sites. Consequently, these eight participants had to be excluded from analysis. There were nine protocol violators who either had a dental cleaning or rinsed with an antimicrobial mouthwash during the study. The data from these nine remained for analysis leaving 51 participants completing the end-of-study assessment (*n* = 24 placebo; *n* = 27 active).

The treatment effect of the active product *versus* placebo on the primary endpoint, BOP, was considered clinically significant with a 48% reduction and approached statistical significance (*p* = 0.056) even with the limited enrollment (Table 3). Significantly reduced BOP from baseline to the end of study was observed in both the active (*p* = 0.001) and placebo groups (*p* ˂ 0.018), although the decrease was almost 2-fold greater in the active group.

**Table 3:** Changes in bleeding upon probing, active versus placebo.

Outcome	Placebo group (*N* = 24)	Active group (*N* = 27)	Between group comparison	Effect size
Reduction in BOP sites	5.83 ± 11.53	11.30 ± 12.92		0.45^d^
	Paired *t*(23) = 2.48	Paired *t*(26) = 4.54	*t*(49) = 1.59	(moderate)
	*p* = 0.021^p^	*p* < 0.001^p^	*p* = 0.060^t^	
% of BOP sites reduction	4.10 ± 7.90	7.91 ± 8.80		0.45^d^
	Paired *t*(23) = 2.55	Paired *t*(26) = 4.67	*t*(49) = 1.62	(moderate)
	*p* = 0.018^p^	*p* < 0.001^p^	*p* = 0.056^t^	

BOP: bleeding on probing.

Note: Data imputed for participants without follow-up (*n* = 4); data excludes super bleeders (*n* = 4).

p Paired sample t-test for the within group comparison;

t Independent samples t-test for the between group comparison;

d Cohen’s d as a measure of effect size

The safety profile of the test product in this study was similar to all previous studies and to real-world experience. There were no related serious adverse events (e.g. hospitalization), and related adverse events were negligible, transient, limited to the oral cavity and did not impede continuation with treatment. Specifically, the most common side effects were a mild, bitter taste, a coating sensation on the teeth and a stinging of the oral mucosa. These reported events diminished from the first application to the final application.

Other important findings about the feasibility of this new model of preventive care were demonstrated. The procedure took 10 to 15 minutes, could be delivered by a dental hygienist or a nurse with minimal training, required no specialized dental equipment, avoided aerosols and most importantly, was well tolerated by the participants.

Nine of 10 participants in the active group rated the treatment ‘very acceptable’, and ‘very important’ in the participant survey (Table 4). Eight in 10 indicated they would likely continue with the coating post study and said it had a positive effect on their general health. In all questions, these responses by the participants in the treated group exceeded those of the participants in the placebo group.

**Table 4:** Participant ratings of the treatment experience as measured by visual analog scale at end-of-study.

Statement	Active group	Placebo group
I found this study’s preventive oral health service to be ... Unacceptable = 0, Very acceptable = 10	9.1	8.5
To me, this preventive oral health service is... Not important = 0, Very important = 10	9.2	8.6
After the study, the chances of my continuing with this preventive oral health service are .... Low = 0, High = 10	8.2	7.6
Since I started this preventive oral health service, the health of my gums has ... Declined = 0, Improved = 10	6.9	6.2
This preventive oral health service has made me feel ... Worse = 0, Better = 10	7.7	6.8
This preventive oral health service has this effect on my general health ... A negative effect = 0, A positive effect = 10	7.9	6.9

## Discussion

The last examiner-based survey of Canadian seniors conducted by the Canada Health Measures Survey (CHMS) in 2007–2009 reported a lower DMFT and a lower prevalence of periodontal disease than found in Ottawa in 2023 (Table 2). While it cannot be said that these 101 seniors in Ottawa represent all Canadian seniors, and thereby that seniors’ oral health has worsened in Canada over the past 15 years, the survey produced surprising and unexpected results. There appears to be a paradox between good dental habits and a significant burden of dental disease. This should concern policymakers as they implement the new seniors’ dental care plan.

Next to hypertension, poor oral health is the most common chronic disease in aging communities [20, 21]. It is also the most expensive disease for many Canadian seniors, and the one where 12% or so, experience fear and anxiety over treatment [22]. The relationship between poor oral health and general health is now accepted as bidirectional, particularly with regard to type 2 diabetes [22], frailty [23], and cognitive disorders [24]. Poor oral health is also considered a modifiable risk factor for cardiovascular disease [25], respiratory disease [26], hypertension [27], malnutrition [28] and systemic inflammation [29]. An adult with moderate to severe periodontal disease has significantly higher scores of serum cholesterol, serum glucose, serum c-reactive protein and systolic blood pressure compared to a peer with good oral health [29]. Moreover, several studies show that adults with poor oral health have significantly more medical visits and hospitalizations than healthy peers [30]. In short, poor oral health puts significant burden on Canada’s healthcare system.

Given this impact of oral health, it is noteworthy that the survey found a high level of dental decay and periodontal disease even in Ottawa seniors who reported good oral hygiene and regular dental visits. This unexpected finding questions the effectiveness of current dental procedures, including home care. The current standard of care for periodontal disease is, as a first step, non-surgical debridement of the tooth surfaces below the gumline. Periodontal scaling is performed by a dental hygienist and in a dental practice.

Periodontal scaling and root planing is considered to be a fundamentally important intervention for periodontitis. Based on various investigations, it has been shown that this nonsurgical intervention leads to improvement in clinical attachment levels by a mean of about 0.6 mm [31, 32]. However, its effectiveness could be reduced in certain populations, particularly those with a chronic disease such as diabetes [33], a condition that is more prevalent in the elderly population, and which was the target population of a consensus report [34]. In addition, scaling and root planing might not be possible in various situations, particularly for patients who are home-bound or institutionalized [35], or where, for example, the generation of aerosols must be avoided. Finally, there is some degree of mild to moderate pain/discomfort in around 60% of patients undergoing scaling and root planing [36, 37], which might also make delivery of this treatment in frail elderly people more difficult. Taken together then, the efficacy of scaling and root planing under routine conditions notwithstanding, other (e.g. aerosol-free, painless etc.) methods for management of periodontal inflammation and dysbiosis as described in this study provide noteworthy benefits. Given that many seniors cannot visit the dentist, not tolerate nor afford periodontal scaling, even the current standard of care is inaccessible.

It is well documented that dental restoration leads to more dental restoration [38–40] Similarly, periodontal infections are often unresponsive to periodontal scaling. One recent Canadian study reported that 1 in 3 sites of BOP were unaffected by scaling [41], while another recent study conducted in the UK over three years reported periodontal scaling had no effect on periodontal disease [42]. This recurrence of dental disease in older adults is largely explained by the continuation of oral dysbiosis after dental treatment [15]. Accordingly, we suggest that more of the same mix of dental services may not address the oral health of Canadian seniors, especially if the barriers to access are not overcome and dysbiosis goes unmanaged.

This Seniors’ Oral Health Study is the first attempt to foster the development of a model of oral health care which could be more suited to millions of Canadian adults who are currently excluded from professional dental care. The study is also the first evaluation of a new preventive treatment which deals with the primary cause of both caries and periodontal disease, and which is delivered by healthcare professionals working with the dental hygienist in an integrated, team approach.

The Study purposively delivered periodontal care in the community without periodontal scaling. Periodontal scaling inherently restricts access to dental care because it is laborious, invasive, can be uncomfortable, generates aerosols, requires frequent repetition, is technique-sensitive and is expensive. By contrast, the procedure reported in this study using 10% chlorhexidine coating removes these barriers. It is non-invasive, requires a fraction of the time, generates no aerosols, and is easy to use by healthcare professionals. Best of all, study participants found it both very acceptable and very important.

The Study also avoided a treatment program which combined the chlorhexidine coating with periodontal scaling. We expected this combination would not be agreeable to the administration, staff and residents in assisted living and long-term care, given the time required for this care, and its generation of aerosols. However, in dental and hygiene practices, this coating is preceded by some periodontal scaling and by some supra-gingival cleaning of the teeth. For example, one hygiene practice has had significant clinical success by graduating the amount and frequency of scaling with the coating according to the level of patients’ risk. By following this protocol, since 2013, this hygienist has reported that hundreds of high-risk seniors have gone for years without caries or periodontal disease [in regular correspondence with Julie DiNardo, RDH; January 2013 to May 2024].

The coating’s treatment effect in this Study is considered to be clinically significant. Compared to the control, there was a 48% reduction in the percentage of sites which bled on probing even when microbial and calcified deposits still remained on the treated teeth. This level of efficacy is similar to the 41% reduction in root caries [43] and 69% reduction in coronal caries [44] in high-risk adults previously reported in controlled studies of this coating. The coating’s efficacy for caries prevention has been approved and recognized to be clinically meaningful by Health Canada (DIN 02046245), the European Medicines Agency (EMA/H/A-29/1258), the United Kingdom (PL 30669) and Ireland (PA1205). The U.S. Food and Drug Administration has defined a clinical meaningful change in oral health as a 20% or more reduction in caries or oral inflammation compared to control [Correspondence and meetings between CHX Technologies and the Division of Dermatological and Dental Drug Products, US Food and Drug Administration under IND 45,466; May 2011 to May 2024]. Moreover, for further context, the only other controlled study in long-term care reported that fluoride varnish reduced caries by only 13% [45].

The Study’s protocol also showed an improvement in the placebo group likely due to a change in oral hygiene behavior, often referred to as the Hawthorne effect which is common in dental studies [46]. In many dental studies, participants tend to brush their teeth more often or more thoroughly, or the caregivers or nursing team may become more attentive to the participants’ oral health, explaining the control group experience of some improvement in oral inflammation over 9 weeks even while receiving only purified water as the therapy. This may be explained by the education provided to Study participants.

## Limitations of the study

1. The treatment and observation lasted 9 weeks which provides a short perspective on a chronic disease. However, real world experience with the effect of the coating in reducing chronic oral inflammation is well established; while receiving intermittent applications of the coating, patients enjoy years of improved oral health. More extended or repeat application periods may result in greater change in oral health.
2. There is a risk of bias due to the small sample and specifically, the sample may not adequately represent the broader population of Canadians, given the fact that the participants were drawn from the Ottawa region, which is a high-income area and where dental insurance is more readily available. Although there were significant decreases seen in the primary outcome in both groups with a near 2-fold larger effect in the active group, recruitment was inadequate to demonstrate statistically significant differences between groups with the outcome potentially also effected by a Hawthorne effect. The recruitment limitation was attributed (i) to the difficulties of conducting controlled studies in older adults [47], particularly with individuals living in long-term care and (ii) to the sponsor’s requirement to report results to the Government of Canada by the fall of 2023.
3. The study was designed to assess specific dependent variables including BOP and did not document microbiological or systemic health markers. Future studies could include these variables for a comprehensive documentation of outcomes.

The Seniors’ Oral Health Study encourages and facilitates more clinical research using controlled and more appropriately, observational study designs on implementing community-based care for high-risk groups. Using the efficacy trends in the Study, the Study statistician has estimated the sample size necessary for more studies in high-risk adults. For observational studies, it is up to 20 participants. Study effect size was used to determine sample size needed for controlled trials. With moderate effect size (Cohen’s *d* = 0.45), 124 individuals will be required in total, equally split with 62 per group.

While there is evidence that chlorhexidine mouthrinses affect the nitric oxide (NO) pathway and stimulate (at least in the short term) hypertension, this study has used a different dosage form of chlorhexidine, a coating which is applied only to the teeth, so as to minimize the antiseptic’s contact with the beneficial anaerobic microorganisms on the dorsum of the tongue which function on the NO pathway. It is also worthy to note that chronic oral inflammation which was reduced by 48% in this time-limited study over about 10 weeks, is as significant to hypertension as age, gender, BMI, income and diabetes [48] in addition to also doubling the risk of high blood pressure in otherwise healthy adults [27]. There may be a tradeoff between preserving beneficial bacteria on the NO pathway to avoid a short term spike in blood pressure, and substantially reducing oral inflammatory load quickly, non-invasively and with full patient compliance to reduce hypertension over the longer term. While this study did not assess the effect on the NO pathway nor on the short or long-term levels of blood pressure, it did report a clinically meaningful reduction in oral inflammatory load which might be considered beneficial to alleviating hypertension. Further research on this tradeoff is needed and future studies should monitor blood pressure changes in the study populations as part of the research protocol.

Follow-up studies with recently hospitalized patients discharged to home and being followed through a home care program, and with high-risk, low-income seniors attending a public health clinic are underway. These studies will evaluate efficacy and feasibility plus the economics and financing of two community-based models of care.

## Summary and conclusions

The Seniors’ Oral Health Study is the first-ever investigation confirming the feasibility of delivering a non-invasive preventive service of chlorhexidine while avoiding periodontal scaling and dental cleaning to seniors where they live and spend their time. Importantly, delivery of the service can be adjunctive for those circumstances where delivery of dental services to high-risk seniors is necessary in the community. The study found an elevated burden of dental disease in a small non-representative sample of Ottawa seniors which was unexpected given the good oral hygiene habits and literacy of participants. This controlled trial illustrated clinically significant treatment effects of this chlorhexidine tooth coating which approached statistical significance compared to a placebo control group, even with very limited enrollment. Study participants rated this community-based oral health service to be very acceptable, very important, worthy of continuation and with very positive effects on general health. This service was delivered quickly in community settings by a hygienist and a nurse with no dental equipment.

A quick, non-invasive, effective and safe procedure which manages the cause of chronic oral disease, and which overcomes barriers to access might facilitate integrated care. Yet, integrated care presumes some fundamental adjustment to the mix and delivery of dental services, especially when there are millions of Canadians who will have subsidized dental care for the first time under the Canada Dental Plan. Serving these millions requires new approaches, more inter-professional collaboration, new thinking and new roles.

## Practical implications

It is logistically possible to deliver a treatment that leads to significant improvement in the degree of oral inflammatory disease in a population of patients who might otherwise be unable to access such care for various reasons.

The provision of treatment using a 10% chlorhexidine coating to teeth leads to important reductions in oral disease (inflammatory as well as dental) substantially reducing the associated burdens.
